# Exploring family, community and healthcare provider perceptions and acceptability for minimal invasive tissue sampling to identify the cause of death in under-five deaths and stillbirths in North India: a qualitative study protocol

**DOI:** 10.1186/s12978-019-0665-1

**Published:** 2019-01-09

**Authors:** Manoja Kumar Das, Narendra Kumar Arora, Reeta Rasaily, Harish Chellani, Harsha Gaikwad, Kathryn Banke

**Affiliations:** 1 The INCLEN Trust International, F1/5, Okhla Industrial Area, Phase 1, New Delhi, 110020 India; 2 Division of Reproductive Biology and Maternal Health, Child Health, Indian Council of Medical Research, Ansari Nagar, New Delhi, 110029 India; 3 Department of Pediatrics, Safdarjung Hospital & Vardhman Mahavir Medical College, New Delhi, 110029 India; 4 Department of Obstetrics and Gynaecology, Safdarjung Hospital & Vardhman Mahavir Medical College, New Delhi, 110029 India; 5 Global Health, Bill and Melinda Gates Foundation, Seattle, WA 98109 USA

**Keywords:** Causes of death, Child, Neonate, Stillbirth, Formative research, Minimally invasive tissue sampling, Autopsy, Family, Health care providers, Community

## Abstract

**Background:**

Around 5.4 million under-five deaths occur globally annually. Over 2.5 million neonatal deaths and an equivalent stillbirths also occur annually worldwide. India is largest contributor to these under-five deaths and stillbirths. To meet the National Health Policy goals aligned with sustainable development targets, adoption of specific strategy and interventions based on exact causes of death and stillbirths are essential. The current cause of death (CoD) labelling process is verbal autopsy based and subject to related limitations. In view of rare diagnostic autopsies, the minimally invasive tissue sampling (MITS) has emerged as a suitable alternate with comparable efficiency to determine CoD. But there is no experience on perception and acceptance for MITS in north Indian context. This formative research is exploring the perceptions and view of families, communities and healthcare providers regarding MITS to determine the acceptability and feasibility.

**Methods:**

The cross-sectional study adopts exploratory qualitative research design. The study will be conducted in New Delhi linked to deaths and stillbirths occurring at a tertiary care hospital. The data from multiple stakeholders will be collected through 53–60 key-informant in-depth interviews (IDIs), 8 focus group discussions (FGDs) and 8–10 death or stillbirth event observations. The IDIs will be done with the parents, family members, community representatives, religious priests, burial site representatives and different health care providers. The FGDs will be conducted with the fathers, mothers, and elderly family members in the community. The data collection will focus on death, post-death rituals, religious practices, willingness to know CoD, acceptability of MITS and decision making dynamics. Data will be analysed following free listing, open coding, selective coding and theme identification. Subsequently 8–10 parents will be approached for consent to conduct MITS using the communication package to be developed using the findings.

**Discussion:**

The study will provide in-depth understanding of the cultural, social, religious practices related to child death and stillbirth and factors that potentially determine acceptance of MITS. The findings will guide development of communication and counselling package and strategies for obtaining consent for MITS. The pilot experience on obtaining consent for MITS will inform suitable refinement and future practice.

## Plain English summary

India tops the list of contributors to childhood and neonatal deaths and stillbirths globally. Each year, about 1 million, 0.8 million and 0.6 million deaths occur before five years, one year and one month of age, respectively, in the country. To meet the National Health Policy and commitment towards sustainable development targets of reducing childhood deaths and stillbirths, it is critical to know the exact causes of childhood deaths and stillbirths. With autopsy occurring rarely and many deaths occurring outside hospitals, the primary method of determining cause of death is use of the verbal autopsy interview. Parents or other close family members are interviewed to collect information that is used to determine the most likely cause of death; however, this method has limitations. Recent work has shown that minimally invasive tissue sampling (MITS) after death offers a more acceptable and feasible alternative to a full autopsy and provides cause of death data that are comparable to what is found with a full autopsy. While there has been some limited work begun in South India using MITS, the level of acceptance and feasibility of MITS in the Indian context among families, communities, and healthcare providers is not known.

This research will explore the views of the families of recently deceased children and infants, community members, and doctors, nurses and support staffs with respect to MITS. The results will identify social, cultural, and other factors that must be taken into consideration when planning to do MITS in New Delhi. This study will be based in a referral hospital in New Delhi and the community around that hospital. It will use a qualitative research design with in-depth interviews and focus group discussions among persons who are selected with the intention to cover a variety of demographic groups, occupations, and viewpoints about acceptance, modality, timing and socio-cultural aspects of the procedure.. This study will provide in-depth understanding of the perceptions, practices and views of families, community members and healthcare providers towards performing MITS among recently deceased children and infants as well as stillborn babies. The information collected will be used to develop an appropriate communication package for approaching and counselling families who have experienced stillbirths or deaths of infants and children at the hospital.

## Background

Despite the significant reduction in childhood mortality globally, approximately 5.4 million under-five children died in 2017, about 15,000 deaths daily [1] [2]. Neonatal deaths constitute about 45% of the under-five deaths [1]. A similar number of stillbirths also occur every year [3, 4]. With its very large population base and high under-five mortality rate, India had about 989,000 under-five deaths during 2017, the highest number of any country. It is estimated that about 1 million under-five, 0.8 million infant deaths and 0.6 million neonatal deaths occurred in India during 2017 [1]. About 0.59 million stillbirths occurred in India during 2015 [5]. The rates of decline in neonatal deaths and stillbirths have been slower compared to decline in post-neonatal deaths [1]. Most of these infant and early childhood deaths are caused by preventable diseases and conditions that could be avoided through cost-effective and basic quality-delivered interventions [1]. The National Health Policy (2017) of India targets reducing under-five mortality from 43 to 23 and neonatal mortality from 24 to ‘single digit’ per 1000 live births by 2025 [6].

To meet these targets and accelerate the reduction in under-five mortality, it is essential to identify the causes of death. In India, like several other low-middle-income countries (LMICs), 21.7–52.6% of the children die without any contact with a skilled healthcare professional [7]. A sizable proportion of those who reach a care provider do not have adequate documentation to identify cause of death (CoD) [7] [8]. Many children are buried soon after death without any investigation into the CoD. Even among those who die at health facilities, the CoD is often difficult to assess, particularly in the case of coexisting illnesses; in addition, the quality and completeness of death certification in facilities is often quite low [9–11]. Complete diagnostic autopsies (CDA), the most comprehensive means to definitively determine CoD, are difficult and not routinely implemented due to sociocultural and religious beliefs, technical, financial and infrastructure limitations [12–16]. Thus most of the causes of death in LMIC settings are based on verbal autopsy (VA), which have several limitations [17–20] . With non-specific signs or symptoms, identifying the exact CoD for neonatal deaths is difficult with the VA method, and it is not useful for stillbirths.

The challenges in existing methods of ascertaining CoD in children often result in uncertainties in national and global disease estimations. The uncertainty about the true CoD compromises effective planning, resource allocation and thereby the effectiveness of the public health policies and programs. In view of the limitations in VA methodology and the impracticality of CDA in LMICs, there is a need for alternative means to determine CoD. Post-mortem minimally invasive autopsy or minimally invasive tissue sampling (MITS), which involves targeted tissue samples from organs for histopathologic and microbiological examinations, offers one approach to improving CoD determination in LMIC settings [21–23]. MITS is quicker, less invasive, more acceptable to families, and less expensive than CDA and studies have shown that CoD results from MITS are comparable to those from CDA [24–26]. However, the perceptions and acceptance of families, communities and healthcare professionals around conducting MITS may vary according to the socio-cultural and religious contexts. A site in South India has recently began implementing MITS, but it is in its early stages and levels of acceptance by families and the community is not yet fully understood [27].

The Child Health and Mortality Prevention Surveillance (CHAMPS) Network supported by the Bill & Melinda Gates Foundation has established field sites to collect robust, standardized, and definitive data on CoD in seven LMIC [28]. CHAMPS aims to determine CoD for all under-five deaths (including stillbirths), capturing both infectious and non-infectious etiologies. CHAMPS uses information from MITS, VAs, and any available clinical information on each death to attribute CoD as precisely as possible. The CHAMPS network includes sites in Africa (Mali, South Africa, Mozambique, Kenya, Ethiopia and Sierra Leone) and one in South Asia (Bangladesh). Selection of one site in India is underway. Following guidance from the Indian Council of Medical Research, a pilot study on MITS is being undertaken in India. The pilot study focuses on assessing the feasibility and acceptability of MITS, capacity building, and standardising sample collection and laboratory procedures before initiating a CHAMPS network site in the country.

### Rationale

Death is a sensitive topic and approaching parents or family members for MITS immediately after a stillbirth or the death of an infant or child is particularly challenging. Conducting MITS in a multicultural and multiethnic Indian setting requires better understanding of the existing norms, beliefs, and practices around death of children and stillbirths. An in-depth understanding of the events and activities done after the death, along with the roles of various family members, is needed to identify how, when, and who should be approached for grief counseling and obtaining consent for MITS. There are few reports on the context, facilitators and barriers for obtaining consent and conducting MITS globally [29–32]. Thus there is need for documenting the family, community and healthcare professionals’ perceptions and views regarding the MITS procedure and existing practices that could influence the acceptability and feasibility in this Indian setting. This qualitative information obtained in this formative research will inform the development of communication materials and strategies to approach the families for MITS in Indian context.

### Study purpose

The formative research has two aims:

1. to understand the personal, family, and community dynamics around grief and associated rituals and cultural practices in the context of stillbirths and deaths of newborns and under-five children in the family in this community in India.
2. to evaluate the feasibility (i.e. acceptability, practicality and implementation), facilitators, and barriers for the proposed use of MITS to identify CoD among stillbirths and under-five deaths.

### Study objectives

We shall undertake the formative research in two phases with specific objectives in each phase as follows:

- *Phase I:* (1.1) to characterize grief and understand the cultural, social, and religious norms, rituals and practices surrounding the death of a child (newborn, infant and child) and stillbirth; and (1.2) to identify the facilitators, barriers and potential opportunities related to conducting MITS and identify key stakeholders in the family, relations and neighborhood who could be engaged to facilitate the MITS.
- *Phase II:* Based on the findings of the phase I; (2.1) to attempt to obtain consent for MITS among a small number of under-five deaths and stillbirths and interview the parents/family members approached to understand their willingness or lack of willingness to participate; and (2.2) to document challenges of obtaining consent for MITS and identify further strategies to overcome these challenges.

## Methods

### Study design

This formative research adopts exploratory qualitative research design including sociological and anthropological approaches, namely ethnography and phenomenology. The data collection techniques to be used include key informant in-depth interviews (IDIs), focus group discussions (FGDs) and observations. The stakeholders for IDIs shall include parents and family members of deceased children or stillbirths, various community members and leaders, religious group representatives, burial site representatives, paediatricians, obstetricians, nurses and other staffs. The FGDs shall be conducted including men and women with under-five children and older family members (decision influencers; mother-in-law and father-in-law). This multi-technique and multi-stakeholder approach will allow synthesis of the data from multiple sources and also help in data triangulation.

### Study setting

The study will be conducted among community members and workers at Safdarjung Hospital (SJH) in New Delhi and will include families who have experienced a stillbirth or the death of an under-five child at SJH. SJH is a tertiary care hospital with 2900 beds and is the largest multispecialty public sector hospital providing care to infants and children in New Delhi.

### Study participants

In phase I, we shall include four sets of populations: (i) parents and family members of stillbirths, neonates, and children who died in SJH in the 6–8 weeks prior to the study; (ii) community members from the SJH catchment area (radius 10–12 km around SJH); (iii) religious leaders and burial site representatives; and (iv) clinicians, health care providers and support staffs from SJH. We will complete 53–60 IDIs with key informants, 8 FGDs with various stakeholders and 8–10 observations of events around under-five deaths/stillbirths in phase I of the study, as detailed in Table 1 below.

**Table 1:** Stakeholders for formative research and sample sizes

Number	Stakeholder category	Sample range
1	Phase 1 (Exploring processes, facilitators and barriers)	
*A*	*In-depth Interviews (IDIs)*	
1.1	Parents and family members (for deaths/stillbirths that occurred 6–8 weeks ago)	
	– Child deaths (> 1 month–5 years)	8–10
	– Neonatal deaths (< 1 month)	8–10
	– Stillbirths	8–10
1.2	Community members (political leaders, elders, key influencers)	4–5
1.3	Religious leaders (Hindu, Muslim, Christian, Sikh)	4
	Burial site representatives (Hindu/Sikh, Muslim, Christian)	3
1.4	Health care providers: Hospital level	
	– Doctors (Pediatrician, neonatologist, obstetrician)	6
	– Nurses (Pediatric, neonatology & obstetrics ward)	6
	– Support staffs (pediatric, neonatology, obstetrics wards/labour room)	6
*B*	*Focus Group Discussions (FGD)* ^a^	
1.5	Focus Group Discussions (FGD)^a^	
	– Fathers of children aged < 5 years	2
	– Mothers of children aged < 5 years	2
	– Father-in-laws (aged > 45 years with grandchildren)	2
	– Mother-in-laws (aged > 45 years with grandchildren)	2
*C*	*Event observations*	
1.6	Event Observations^b^	
	– Neonatal and child deaths occurring in hospital	4–5
	– Stillbirths occurring in hospital	4–5
2	Phase II (Obtaining consent for MITS)	
2.1	Family members of deaths/stillbirths^c^	
	– Child death (> 1 month–5 years)	3
	– Neonatal death (< 1 months)	3
	– Stillbirths/intrauterine deaths	4

^a^The participants for FGD shall not be from the households with recent death of child or stillbirth

^b^The events for observation shall include deaths/stillbirths occurring in the hospital and the observations shall be done from the time of the declaration of the event (death/stillbirth) till departure of the body and family members from the hospital

^c^The family members of deaths (child under five years age/neonates) or stillbirths occurring at the hospital shall be approached for MITS

For the phase II, the stakeholders who shall participate include parents and family members of recent stillbirths and under-five deaths and clinicians and healthcare providers at SJH. Using the communication documents and approaches developed from the findings in phase I, parents and family members of 6 neonatal/child deaths and 4 stillbirths occurring at SJH shall be approached and asked for consent to perform MITS. This practical experience will provide an understanding of the factors related to acceptance and/or refusal of MITS.

#### Selection of the participants

For selection of the stakeholders in phase 1, we shall adopt the following processes.

- *Parents and family members of children died or stillbirths:* The families who experienced child death and/or stillbirth at in the hospital shall be shall be contacted at their residence 6–8 weeks after the event. The families with neonatal and child deaths and stillbirths from different religions and geographies where both parents are available shall be purposively identified and approached for consent. The research team shall be in close contact with the hospital staff to identify the deaths. The research team shall contact the families after 6–8 weeks of death to assess the status of the parents and family members. If found stable for interaction and interview, the staff shall explain about the study, related processes and seek consent for the interview.
- *Community members:* The community level participants (political leaders, elders, and key influencers) for IDIs and stakeholders for FGDs shall be identified by the research team through visit in the community and enquiry.
- *Religious leaders:* The priests from different religions (Hindu, Muslim, Christian and Sikh), who assist in death-related rituals shall be purposively identified from the catchment area.
- *Burial site representatives:* The representatives from cemeteries for different religions (Hindu/Sikh, Muslim and Christian) shall be purposively identified from the catchment area.
- *Health care providers:* From the hospital, various healthcare providers shall be identified. The treating doctors (pediatrician, neonatologist and obstetrician) from different levels, trainee residents and specialists, nurses and supporting staffs engaged in different units shall be selected purposively.
- *Observations of events at hospital:* The deaths of children and neonates and stillbirths occurring in the hospital shall be observed.

The families and stakeholders from Delhi only shall be identified considering the logistics feasibility. The families of deceased children or stillbirths with incomplete address and outside Delhi shall be excluded. A community mobilisation team shall enable effective engagement with the community and trust building for selection, explanation and consent form data collection, in view of the sensitivity nature of the issue. Informal discussion and snowballing techniques shall be used to undertake the community stakeholder mapping.

For the phase II, selection of the stakeholders shall follow the following process.

- *Approaching for consent for MITS:* The parents and family members of a convenience sample of 3 neonates, 3 children under five, and 4 stillbirths occurring at the hospital shall be approached immediately by the MITS team after death at the hospital. The formative research team shall support the MITs team from the hospital. These deaths and stillbirths shall be identified with help from the treating doctors.

### Data collection

#### In-depth interviews (IDIs)

We will identify the suitable community stakeholders as mentioned above and invite them to participate in the study through home/community visit. The religious leaders and burial site representatives shall be visited at their place of work or residence. The IDIs shall be held at locations preferred by the stakeholders ensuring appropriate privacy, household or community level for non-hospital stakeholders and at SJH for hospital staffs. Written informed consent will be obtained along with permission for audio recording of the interview. At least two research team members trained in conducting qualitative IDIs shall conduct the IDIs. One member will be the lead interviewer and the other will be the note-taker. The IDIs with parents and family members of deceased children/stillbirths will initially involve discussion around any illnesses/signs/symptoms that the child experienced (or that the mother experienced during her pregnancy for stillbirths), the hospital course, and their experience with and interactions with healthcare providers prior to the death (including communication with/by healthcare providers). Then the discussion will focus on the experience around the death/stillbirth and post-death/stillbirth procedures, grief, rituals, and coping mechanisms. Finally the interview will explore the perceptions about causes of death, desire to know cause of death/stillbirth, acceptability of MITS procedure, decision making dynamics, and facilitators and barriers for consent to conduct MITS. The IDIs with religious leaders and burial site representatives shall focus on the community’s perceptions and practices around deaths of under-five children and stillbirths, the acceptability of MITS from a religious and cultural perspective, and how to approach families. The IDIs with hospital staffs will focus on care of children/pregnant women, communication and decision making during hospitalization of children under five or cases of stillbirth, death declaration, causes of death/stillbirth at SJH, potential acceptability and conduct of MITS from the staffs’ perspectives, and the potential facilitators and barriers for being able to obtain consent and perform MITS at SJH. IDIs shall be conducted using interview guides with less structured, open-ended questions addressing the key domains identified by literature review and discussion with expert groups. Any new domain identified during the IDI shall be further pursued. We anticipate that the planned number of IDIs shall be capture the issues under study, but additional IDIs may be conducted if needed till data saturation is achieved. Each IDI will be audio recorded apart from field notes taken by the note-taker. Each IDI is expected to last for 45–60 min.

#### Focus group discussions (FGDs)

The FGDs shall be done with the father, mother of under-five children and elder family members (fathers-in-law and mothers-in-law), who have not recently experienced child death or stillbirth to capture the community level perceptions, practices and opinions about death, willingness to know causes of death and acceptance of MITS. The FGDs shall discuss initially about the illnesses in children, care during pregnancy, practices related to child death and stillbirth, grieving and coping mechanisms, their perceptions about the causes of child deaths and stillbirths, interest in knowing the cause of death for children/stillbirths, acceptability of MITS and perceived facilitators and barriers for MITS among under-five deaths and stillbirths. The FGDs will be conducted at convenient and neutral venues in the community. Each FGD will involve 8–10 participants from similar category. The FGDs will be facilitated by one experienced senior team member supported by two note-takers and one for sociogram. Written informed consent from the FGD participants will be obtained before the FGD for participation and audio-recorded. A FGD guide with domains shall be used to conduct the discussions. We plan to conduct eight FGDs, but additional FGD may be done if needed for data saturation.

#### Observations of death/stillbirths

The research staff shall obtain informed consent from a convenience sample of families that experience under-five deaths and stillbirths at SJH. A team member will observe the events and communication exchanges between the hospital staffs and family members, including verbal and non-verbal expressions, from the time of consent (shortly after the death/stillbirth) until the family is discharged from the hospital. The research staff shall only observe to document the flow of events, activities by different stakeholders and communication (verbal and non-verbal) exchanges. No interview or audio-recording shall be done for the observation. Notes shall be taken by the study team using a time-event-activity sequence matrix.

#### Preparation for grief counseling and obtaining consent for MITS

Based on the findings from various data collected during phase I including IDIs, FGDs, observations and literature, communication materials and tools shall be developed for grief counseling and the approach to obtain consent for MITS, keeping the religious and sociocultural practices in context. The MITS team at hospital shall be oriented on the findings and various aspects of grief counseling and communication to obtain consent for MITS.

#### Approach for MITS

During phase II, the MITS team from SJH shall approach the families of recent death and stillbirths (after declaration death/stillbirth) to obtain consent to undertake MITS. The explanation process, questions asked, concerns expressed, responses given, behavior and expressions of different members shall be documented by the research team.

#### Data management

During the IDIs, FGDs and observations, the note-taker(s) shall make field notes and capture the expressions and key statements by the respondents. The IDIs and FGDs will be transcribed verbatim in local language using the audio-record followed by translation into English. Another team member shall conduct quality check of the transcription referring to the audio-record to ensure completeness and correctness. A senior study team member will perform quality checks of transcripts and translations to ensure completeness and correctness. Field notes taken during IDIs, FGDs and observations will also be transcribed. The data shall be entered using the INCLEN Qualitative Data Analysis Software (IQDAS), which allows data entry, organization and retrieval for data for analysis in English and Indian languages. The data entered shall be checked for correctness and completeness by the study staff. The data shall be accessible to only authorized study team members. The data entered shall be saved into the server and backed up on daily basis.

#### Ethical considerations

The research team will explain the study to participants and will ask them to give informed, written consent prior to participation in any aspects of this study. For any illiterate participants, the explanation will occur in the presence of a witness and thumbprints will be used to indicate their consent to participate. Confidentiality and anonymity of participants will be assured. The protocol was reviewed and approved by the ethics committees of the participating institutes prior to initiation of this study. There will be no direct financial benefit or compensation for the participants.

#### Data Analysis

Data analysis will be done inductively following the steps: free listing, domain identification, coding, and cross tabulation. The transcript reading shall undergo through a reiterative process to identify the emergent themes, which shall be coded, compared, contrasted and refined. The analysis process will involve an iterative process where data are coded, compared, contrasted and refined to generate emergent themes. From the transcripts the team shall identify relevant text segments (free listing) which will be later summarized and labeled with a code (open coding). These codes will be then compared for identifying the connections between categories (axial coding) and grouped into fewer categories (selective coding). Categories with similarity will be further assembled under key themes. Two study team members will undertake the free listing, coding, category creation, and thematic analyses, and discrepancies will be resolved to minimize bias. The emerging themes from different stakeholders (mothers, fathers, family members, religious groups, community members, and healthcare providers) and data collection methods (IDIs and FGDs) will be compared to identify similarities, differences and any inconsistencies [33].

## Discussion

This formative research will provide in-depth information about: [1] the cultural, social, and religious norms, rituals and practices related to child death and stillbirth in north-Indian and primarily urban context; [2] the family attitudes, beliefs, practices and perceptions related to child deaths and stillbirths; [3] the healthcare providers’ perceptions and practices related to communication and interactions with families around under five deaths and stillbirths, causes of death, and MITS; and [4] the interest of families in knowing the causes of death, as well as the facilitators, barriers, and potential opportunities and strategies for obtaining consent for MITS in this setting. This study will also provide the initial experience of seeking consent for MITS in a hospital setting, which will be used to develop and refine a communication and counselling package and strategies to be implemented in the following MITS pilot study at SJH. The findings will have implications for potential adoption of MITS as a new practice for more precise cause of death determination in India.
